# Dedicated Vertical Physician Pilot in a Rotational Patient Assignment Emergency Department

**DOI:** 10.5811/westjem.64648

**Published:** 2026-11-01

**Authors:** Nicole R. Hodgson, Jessica Monas, Aaron Levy, Chrissie VanLandschoot, Mark Dollar, Benjamin Phelps, Andrej Urumov

**Affiliations:** Mayo Clinic Arizona, Department of Emergency Medicine, Phoenix, Arizona

## Abstract

**Introduction:**

Emergency department (ED) vertical patient pathways (VPP) assign patients to a dedicated care area with chairs, avoiding the use of traditional ED beds. The effect of reassigning a dedicated vertical emergency physician (EP) onto an established rotational patient assignment (RPA) system, which prospectively assigns arriving patients to EP queues, is unknown. We sought to describe operational outcomes and staff satisfaction with trialing this role.

**Methods:**

We conducted a single-center, hypothesis-generating exploratory pilot at a 56-bed tertiary-care ED in which one 8.75-hour EP shift on five Mondays was repurposed into an 8.75-hour dedicated VPP role within a chair-based vertical patient care area while the rotational patient assignment system remained in place; three prior comparable Mondays served as comparison. Primary outcome was discharged-patient ED length of stay (LOS); secondary outcomes included VPP upgrades to a bed, patient numbers seen by the dedicated shift, and staff satisfaction. We report descriptive analyses.

**Results:**

We analyzed 529 discharged-patient encounters: 199 (37.6%) during the preintervention period and 330 (62.4%) during the postintervention period. Median ED LOS for discharged patients did not demonstrate a consistent directional change, decreasing by seven minutes (3.1%) in the overall ED (226 vs 219 minutes) and increasing by 18.5 minutes (13.0%) among patients initially evaluated via the VPP (142.5 vs 161 minutes). The dedicated VPP EP saw three more patients per shift (21.4%; median 17 vs 14), and VPP upgrades fell from 15/68 (22.1%) to 5/130 (3.8%). Timeliness and communication were rated favorably by 4/4 VPP physician respondents and 10/12 and 16/17 registered nurse/allied health respondents, respectively; 3/4 VPP physician respondents vs 1/29 non-VPP physician respondents supported continuing the model.

**Conclusion:**

Reassigning an existing EP to a dedicated vertical EP role within an ongoing rotational patient assignment system was not accompanied by shortened discharged-patient length of stay. Fewer VPP upgrades and a modest increase in patients seen by the dedicated shift were observed, but patient selection and acuity limit interpretation. Staff reported favorable teamwork within the vertical space, whereas non-VPP physicians reported less manageable workloads and little support for continuing the model.

## INTRODUCTION

Vertical patient pathways (VPP) stream lower-acuity, ambulatory emergency department (ED) patients into nontraditional chair-based spaces, reserving traditional beds; prior reviews report favorable throughput effects.^1^–^3^ To our knowledge, no published study has evaluated a dedicated vertical emergency physician (EP) within an established rotational patient assignment (RPA) system. The RPA system prospectively assigns arriving patients to EP queues, making responsibility explicit from patient arrival and distributing workload prospectively.^4^,^5^

A dedicated vertical EP might accelerate care for ambulatory patients and preserve monitored beds, but removing lower-acuity cases from the general queue could redistribute workload or create handoffs when patients require a main ED bed. In this hypothesis-generating pilot, we introduced a dedicated vertical EP while RPA otherwise remained in place and described discharged-patient length of stay (LOS), vertical upgrades, shift volume, and staff satisfaction.

## METHODS

The study ED is a 56-bed tertiary care center serving approximately 60,000 patients yearly. A computerized RPA system assigns patients to an EP upon arrival and stops assignments either two hours before shift end or after 17 patients, whichever comes first.^5^ Previous work at our facility helped define a VPP for patients who may be cared for without a traditional ED bed.^6^ Triage registered nurses (RNs) slate ambulatory patients with Emergency Severity Index (ESI) scores of 4, 5, or ESI 3 skin and ocular complaints for VPP, while collaborating with assigned EPs to route other appropriate patients. The VPP assessment area adjoins the main ED and contains five curtained reclining chair bays and a charting area. Vertical RNs alert the assigned EP to a VPP patient awaiting assessment.

On five Mondays from February 2 to March 2, 2026, one EP shift that normally received assigned patients from 7:45 am to 2:30 pm was repurposed as a dedicated VPP-physician shift. This physician sat in the vertical area and attempted to safely manage as many vertical patients as possible. Automatically eligible patients included ESI 4–5 and ESI 3 skin or ocular complaints, with additional patients chosen at physician discretion. The VPP EP stopped receiving patients at 2:30 pm but could choose to take additional patients until 4:30 pm. No additional RN or technician staffing or preferential resource access was added during the pilot.

Our ED uses no physician assistants or nurse practitioners, nor does it staff a triage EP. Rotating residents work one-to-one with EPs and see approximately 15% of encounters; residents perform VPP assessments concurrently with attendings for efficiency. The dedicated VPP EP was not assigned a learner.

Three Mondays in January 2026 (January 12, 19, and 26) were selected as comparison dates due to similar physician staffing and study hour census. Analyses excluded admitted patients to limit confounding from inpatient capacity constraints. Primary outcome was discharged-patient ED length of stay (LOS) for arrivals from 7:45 am to 2:30 pm, calculated from electronic health record (EHR) arrival and discharge timestamps. Secondary outcomes included VPP upgrades, numbers of patients evaluated by the dedicated shift, and staff survey responses. Encounter assignment and upgrade data were obtained from the EHR. We summarized encounter characteristics descriptively with medians (interquartile ranges), means (standard deviations [SD]), and proportions. The pilot schedule determined study size, and no formal sample size calculation was performed. As a hypothesis-generating pilot, we did not perform hypothesis testing or calculate confidence intervals.

**Population Health Research Capsule**

What do we already know about this issue?

*Vertical pathways can improve emergency department (ED) flow, but assigning a dedicated physician within a rotational patient assignment model has not been evaluated.*

What was the research question?

*What were the operational outcomes and staff perceptions during a dedicated vertical physician pilot?*

What was the major finding of the study?

*Discharged-patient length of stay decreased 3.1% (226 vs. 219 minutes) in the overall ED and increased 13.0% (142.5 minutes without vs. 161 with a dedicated physician) among vertical patients.*

How does this improve population health?

*These findings help ED leaders design department flow that preserves timely access while anticipating workload tradeoffs.*

We requested anonymous feedback from EPs working on trial Mondays and the following Tuesday, which retained the same staffing but moved the VPP physician back into RPA. Each of the five trial Mondays was staffed by a different VPP physician, and all five VPP physicians submitted a survey. Nursing leadership conducted a similar survey for RNs and allied staff who played a role in the VPP workflow during trial Mondays (VPP-assigned, triage and navigator RNs, VPP-assigned technicians, and health unit coordinators). Appendix 1 contains survey language.

We queried staff regarding workload manageability, timeliness, communication, and support for continuing the VPP physician role. We asked physicians whether transitions of care felt safe and RNs and allied staff whether VPP inclusion criteria were clear. All surveys included an open-ended response option. Survey results were summarized using item-level denominators, with incomplete responses excluded.

The Mayo Clinic Institutional Review Board provided an exemption from full review. We received no external funding. We report the work in accordance with Standards for Quality Improvement Reporting Excellence (SQUIRE) 2.0 guidance.

## RESULTS

Table 1 summarizes encounter characteristics and operational results.

Table 2 summarizes survey responses.

Per-day ED arrivals during trial hours were nearly identical between periods (baseline 101.7/day vs trial 101.6/day). During the baseline period, 22.3% of ED arrivals were initially roomed vertically, compared with 25.6% during the trial. On baseline days, the comparison physician shift evaluated a median of 14 patients daily (range 14–16). On trial days, the VPP EP initially evaluated a median of 17 patients daily (range 13–22). Among patients initially evaluated vertically, trial patients were younger (median 64.5 vs 73 years) and less often required transfer to the main ED (“VPP upgrades”; 5/130 [3.8%] vs 15/68 [22.1%]). During the trial, each upgrade required transition of care from the VPP physician to another main EP. During the baseline period, the initial EP retained care.

Across open-ended EP comments, recurring themes included friction related to patient selection and the RPA workflow (disruption when charts were reviewed by a main ED EP before VPP reassignment and concerns about inequitable distribution of higher-acuity patients), as well as VPP area workspace and privacy limitations. The non-VPP EPs emphasized perceived loss of lower-acuity patients and downstream effects on workload complexity and timing of patient assignments, while VPP EPs described challenges of remaining physically embedded in the vertical space while managing sporadic inflow and care transitions. In contrast, RN and allied staff comments highlighted improved communication with a single dedicated physician, perceived reductions in delays, and stronger teamwork within VPP.

## DISCUSSION

In this limited, hypothesis-generating pilot, repurposing one physician shift into a dedicated VPP role within an otherwise unchanged RPA system was not accompanied by a consistent directional change in discharged-patient LOS. The dedicated VPP physician evaluated slightly more patients, and fewer patients required upgrades. Because acuity-adjusted workload, resource use, and disposition complexity were not measured, the difference between 17 and 14 patients should not be interpreted as an efficiency gain.

The observed pattern may reflect differences in vertical-patient selection more than changes in department-level throughput. The lower upgrade rate and younger age of VPP patients may reflect more selective triage. Prior ED work suggests clinician choice may favor simpler presentations.^7^ Under the usual workflow, physicians retain ownership if a vertical patient needs a bed; during the pilot, an unsuccessful vertical trial required a handoff, possibly discouraging borderline selection. The RPA system provides prospective ownership, while the pilot added a physician-to-physician transfer for each upgrade.^5^ Handoffs may contain omissions or discrepancies; this may have affected physician behavior or acceptance.^8^–^10^ Day-to-day case mix, physician variation, or colocation allowing continued chair observation could also contribute. Because the data did not distinguish automatic from discretionary selection, we cannot determine the explanation.

Staff perceptions diverged sharply, with support for role continuation at 3/4 among VPP physician respondents and 1/29 among non-VPP physicians; however, the small, asymmetric denominators preclude true statistical comparison. Physicians assigned to the vertical pathway, RNs, and allied staff reported favorable local teamwork, whereas non-VPP physicians reported less manageable workloads. Removing lower-acuity patients from the general pool may have left a more complex assignment mix.

To our knowledge, this is the first report of a dedicated vertical EP within an established RPA system. The resource-neutral pilot cannot establish effectiveness, but it addresses a design question relevant to ED medical directors. Future studies should evaluate acuity-adjusted workload, resource use, and handoff burden.

## LIMITATIONS

This single-center pilot included only eight morning study days, relatively low VPP volume, and five unique VPP physicians. Item-level missingness reduced some VPP physician denominators to two or four. Analyses were descriptive and limited to discharged patients arriving during study hours. We did not assess admission rate, door-to-disposition time for all ED patients, imaging, laboratory or consultation use, acuity-adjusted workload, or automatic versus discretionary selection. Individual-physician variation may have affected findings; results may not generalize to other time periods or EDs with different staffing, volumes, or assignment models.

## CONCLUSION

In this pilot, adding a dedicated vertical EP to an existing rotational patient assignment system was not accompanied by a consistent directional change in discharged-patient length of stay. Fewer upgrades and slightly higher dedicated-shift volume were observed, but patient selection and acuity limit interpretation. Physicians assigned to the vertical pathway, RNs, and allied staff viewed teamwork favorably, whereas non-VPP physicians described less manageable workloads and rarely supported continuation. Emergency department leaders should balance local teamwork and communication against workload redistribution and handoff burden.

## Supplementary Information



## Figures and Tables

**Table 1:** Descriptive encounter and operational characteristics for discharged patients before and during a pilot of a dedicated vertical physician in an emergency department with rotational patient assignment.

Characteristic	All ED Preintervention (N = 199)	All ED Postintervention (N = 330)	Vertical Preintervention (n = 58)	Vertical Postintervention (n = 117)
Sex female (%)	55%	51%	56%	46%
Age median years (IQR)	66 (48–78)	66 (49.25–77)	73 (55.5–80)	64.5 (44–76)
Race White (%)	92%	89%	91%	89%
ESI mean (SD)	3.03 (0.61)	3.10 (0.61)	3.5 (0.56)	3.59 (0.52)
ED median length of stay minutes (IQR)	226 (161.5–295.5)	219 (159–294)	142.5 (100–255)	161 (106–221)

*ED*, emergency department; *ESI*, Emergency Severity Index; *IQR*, interquartile range; *SD*, standard deviation.

Table 1 is restricted to discharged patients; upgrade denominators include all patients initially evaluated in the vertical patient pathway.

**Table 2:** Survey results (favorable responses on five-point Likert items) in a pilot of a dedicated vertical physician in an emergency department with rotational patient assignment.

Survey item	VPP physicians (trial days)	Non-VPP physicians (trial days)	Usual schedule physicians (typical days)	RNs and Allied Health (trial days)
Survey submissions, *N*	5	29	18	17
Perceived manageability of patient load	2/4 (50%)	9/29 (31%)	15/18 (83%)	15/15 (100%)
Timeliness (able to see patients in a timely fashion)	4/4 (100%)	10/29 (34%)	11/18 (61%)	10/12 (83%)
Communication and coordination (able to contact RNs or able to effectively communicate with assigned physician)	4/4 (100%)	21/27 (78%)	17/18 (94%)	16/17 (94%)
Transitions of care felt safe	1/2 (50%)	3/6 (50%)	11/13 (85%)	—
Support continuing dedicated vertical physician role	3/4 (75%)	1/29 (3%)	1/15 (7%)	13/17 (76%)
(RNs and allied health) Clear understanding of which patients should be in the vertical space	—	—	—	14/14 (100%)

Values shown are favorable responses, n/N (%), based on item-level responses (missing responses excluded); denominators differ from total survey submissions because respondents could skip items. Likert scale: 1 = Strongly disagree to 5 = Strongly agree. “—” indicates item not applicable. Favorable responses were defined as Agree/strongly agree, except for the workload item in physician surveys, which was inversely worded (favorable = disagree/strongly disagree).

*RN*, registered nurse; *VPP*, vertical patient pathway.
