# Supplementary material for: Dedicated Vertical Physician Pilot in a Rotational Patient Assignment Emergency Department
Source: West J Emerg Med. 2026 Nov 1;27(6):1758–62. doi: 10.5811/westjem.64648 (PMC13761713; doi:10.5811/westjem.64648)
Supplement: Supplementary file 1 [file wjem-27-1758-s001.docx]

**Appendix 1. Survey Instruments**

This appendix lists the questions administered to each respondent group during the Dedicated Vertical Patient Pathway (VPP) Physician pilot. Unless otherwise specified, Likert-scale items used the response anchors: Strongly disagree, Disagree, Neutral, Agree, Strongly agree.

**A. Physician Surveys**

*All physician surveys included five Likert-scale items and one free-text item.*

**A1. Vertical Patient Pathway Physician Survey (Trial Days)**

*Administered to the physician assigned to the vertical zone (VPP physician) on trial days.*

1. I struggled to manage my patient load today.

2. I was able to see my patients in a timely fashion.

3. Transitions of care to the main ED physicians felt safe.

4. I could easily contact the RNs caring for my patients to coordinate care.

5. I would like to see the dedicated vertical physician role continue on future shifts.

6. Any additional comments that you would like to share? (Free text)

**A2. Non-Vertical Patient Pathway Physician Survey (Trial Days)**

*Administered to physicians working on trial days, excluding the VPP physician and overnight shifts.*

1. I struggled to manage my patient load today.

2. I was able to see my patients in a timely fashion.

3. Transitions of care from the VPP physician to myself felt safe.

4. I could easily contact the RNs caring for my patients to coordinate care.

5. I would like to see the dedicated vertical physician role continue on future shifts.

6. Any additional comments that you would like to share? (Free text)

**A3. Usual Schedule Physician Survey (Typical Days)**

*Administered on typical schedule days (all shifts except overnights).*

1. I struggled to manage my patient load today.

2. I was able to see my patients in a timely fashion.

3. Transitions of care from another physician to myself felt safe.

4. I could easily contact the RNs caring for my patients to coordinate care.

5. I would like to see the dedicated vertical physician role continue on future shifts.

6. Any additional comments that you would like to share? (Free text)

**B. Allied Health Staff Survey (Trial Days)**

*Administered to non-physician staff assigned to VPP-related roles on trial days (VPP-assigned RNs, VPP-assigned ED technicians, Triage RNs, Navigator RNs, and Health Unit Coordinators [HUCs]).*

1. What was your role in the Dedicated VPP Pilot today? (Select one: VPP RN; VPP Tech; HUC; Triage RN; Navigator)

2. I had a clear understanding of which patients were appropriate for the Vertical Space.

3. I was able to manage my assigned patient load effectively today.

4. I was able to see my patients in a timely manner.

5. I was able to effectively communicate with the dedicated VPP provider.

6. I would like the dedicated vertical physician role to continue in the future.

7. Describe any successes you experienced in Vertical Space with a dedicated provider: (Free text)

8. Describe any opportunities for improvement regarding Vertical Space or the dedicated-provider workflow: (Free text)
